# UV-induced Zn:Cd/S quantum dots in-situ formed in the presence of thiols for sensitive and selective fluorescence detection of thiols

**DOI:** 10.1038/s41598-021-93137-4

**Published:** 2021-07-05

**Authors:** Navid Assi, Lukas Nejdl, Kristyna Zemankova, Kristyna Pavelicova, Jaroslava Bezdekova, Mirek Macka, Vojtech Adam, Marketa Vaculovicova

**Affiliations:** 1 Department of Chemistry and Biochemistry, Mendel University in Brno, Zemedelska 1, 613 00 Brno, Czech Republic; 2 Central European Institute of Technology, Brno University of Technology, Purkynova 123, 612 00 Brno, Czech Republic

**Keywords:** Diagnostic markers, Bioanalytical chemistry, Fluorescent probes, Medical and clinical diagnostics

## Abstract

In this work, we explored a new approach to a simple and sensitive fluorescence detection of thiols. The approach takes advantage of an in-situ formation of UV light-induced fluorescent nanoparticles (ZnCd/S quantum dots), while utilizing the thiol group of the analyte as a capping agent. The selectivity is ensured by the selective isolation of the thiol analyte by a polydopamine molecularly imprinted polymeric (MIP) layer. Based on this approach, a method for determination of thiols was designed. Key experimental parameters were optimized, including those of molecular imprinting and of effective model thiol molecule (l-cysteine) isolation. The relationship between the fluorescence intensity of ZnCd/S quantum dots and the concentration of l-cysteine in the range of 12–150 µg/mL was linear with a detection limit of 3.6 µg/mL. The molecularly imprinted polymer showed high absorption mass capacity (1.73 mg/g) and an excellent selectivity factor for l-cysteine compared to N-acetyl-l-cysteine and l-homocysteine of 63.56 and 87.48, respectively. The proposed method was applied for l-cysteine determination in human urine with satisfactory results. Due to a high variability of molecular imprinting technology and versatility of in-situ probe formation, methods based on this approach can be easily adopted for analysis of any thiol of interest.

## Introduction

Molecular imprinting technology is an approach combining benefits provided by polymer chemistry, material chemistry, biochemistry, and other scientific areas. Molecularly imprinted polymer (MIP) is a three-dimensional cross-linked structure enabling unique recognition ability^1^. Compared to conventional recognition elements—antibodies, MIPs possess a much wider range of advantages including a good plasticity, a simple way of preparation, highly selective interactions with the analyte, and low costs. MIPs have found numerous applications in different fields such as solid-phase extraction, chromatographic separations, chemical biomimetic sensing, and even enzyme-mimetic techniques^2,3^. Common monomers used for MIPs preparation include acrylamides, methacrylates, or ethoxysilanes. Besides these monomers, initiator-free polymerization of dopamine has the potential to provide numerous benefits such as effective coverage of various surface materials or simple oxidative self-polymerization. Dopamine contains catechol and amine functional groups, which can be polymerized under alkaline conditions to form a polydopamine layer. Among the important features of polydopamine is its ability to create covalent and non-covalent interactions with templates/analytes^4,5^.

Thiols (e.g. l-cysteine, glutathione or γ-glutamyl-cysteine) play very important role in the human organism^6^ and therefore, the here presented approach aims at the detection of these molecules. l-cysteine was selected as the model analyte in order to demonstrate the approach, which can be simply adjusted also for the detection of other thiols. l-cysteine, a sulfur-containing and non-essential amino acid, has a great significance in biological systems, especially in immune cells and a variety of fundamental physiological processes such as metabolism, protein synthesis and/or detoxification reactions^7,8^. Deficiency as well as elevated level of l-cysteine have been associated with liver damage^9^, cervical cancer^10^, hematopoiesis decrease^11^, neurotoxicity^12^, and other pathological conditions including HIV^13^. For example, cystinuria is a genetic disorder related to *SLC3A1* and *SLC7A9* genes. In this disease, cystine stones are formed when the cystine content in urine exceeds 300 mg/L at pH between 4.5 and 7.0^14^. Hence, l-cysteine determination is very important and its detection has been reported using separation techniques such as HPLC and capillary electrophoresis^15^, spectrophotometry^16^, chemiluminescence^17^, and electrochemistry^18^. Most of these techniques have drawbacks and inconveniences such as often complicated sample preparation or insufficient sensitivity, and low selectivity^19,20^.

Moreover, l-cysteine has a very low molar absorptivity as it lacks suitable chromophores, therefore fluorescence-labeling via thiol groups is a common technique used in diagnostics^21^. Besides fluorescent labeling tag based on organic fluorophores, quantum dots (QDs) have recently gained substantial attention. Their applications and properties depend on the molecule stabilizing the core—capping agent. The capping agent is one of the most important implements to control the particles size and agglomeration. It surrounds the nucleated particles and limits the particles size. Thiol groups are the most conventionally preferred ligands for stabilizing the QDs with reduction the toxicity and making them hydrophobic. The energy levels of the thiol inhibit hole trapping and the resultant material is highly fluorescent in nature^22^. Surface capping of QDs most often takes advantage of surface interactions between a thiol-molecule and the metal/metalloid core of the QD nanocrystal. Importantly, specific interactions between the capping agents and targeted molecules may be subsequently exploited for thiol detection^23^. Synthesis of QDs usually requires relatively demanding procedures which need to be followed by a relatively complicated conjugation procedure in case if QDs are utilized as fluorescent labels.

In our previous studies, we have demonstrated that QDs can be easily synthetized from a mixture of metal ions and thiols using irradiation by UV light^24^. The amount of QDs formed and their fluorescence intensity is proportionate to the thiol concentration. Therefore, in this study, for the first time we show that thiols can be easily quantified based on the resulting fluorescence signal.

Herein, we focused on direct fluorescent detection/determination of thiols, here demonstrated by the detection of l-cysteine. The overall idea rests in combining a selective isolation of l-cysteine by a MIP layer with its subsequent fluorescence detection using in-situ formed fluorescent probes (ZnCd/S QDs). It is noteworthy that in this approach l-cysteine is not only the analyte but also serves as the capping agent at the surface of UV light-induced probes ZnCd/S QDs).

## Materials and methods

All methods and protocols were carried out in accordance with guidelines and regulations of Mendel University in Brno, urine samples were voluntarily provided by the authors of the study after their informed consent. No underage persons were involved. The methods and experimental protocols were approved by ethical committee of Mendel University in Brno.

### Chemicals and reagents

All chemicals were purchased from Sigma-Aldrich (St. Louis, MO, USA) in ACS purity. MilliQ water was used throughout the experiments.

### Preparation of MIP

MIPs were prepared by self‐polymerization of dopamine hydrochloride as a monomer using l-cysteine as a template. Dopamine hydrochloride and l-cysteine were mixed (in the optimized ratio) in of Tris–HCl buffer (100 mM, pH 8.5), 50 µL of the mixture was pipetted into the UV-transparent 96-well plate with a flat bottom by CoStar (Corning, USA), and polymerized at ambient temperature overnight. Non-imprinted polymers (NIPs) were procured by the same procedure in absence of l-cysteine.

### Test sample analysis procedure

After MIP preparation, its efficiency was evaluated by analysis of test samples (l-cysteine standard solution). For this intent, the dark brown surface layer of the MIP/NIP was washed twice with 50 µL of optimized washing solvent to remove the template. These prepared MIPs/NIPs were used for subsequent experiments. The first step of the analysis is the sample loading; 50 µL of l-cysteine standard solutions (at various concentrations) were added to the well plate and interacted with MIP/NIP for 1 h at 250 rpm at ambient temperature. Next, the sample solution was discarded and the polymer layer was washed with 20 µL of water. The second step involved in-situ formation of fluorescent probes (ZnCd/S QDs) according to our previous work^25^. Briefly, 50 µL of mixture of precursors (zinc acetate (0.53 mM) and cadmium acetate (0.082 mM) prepared in phosphate buffer (100 mM, pH 7.0)) was added to the MIP/NIP after sample loading. The plate was irradiated in the UV transilluminator (Vilber Lourmat, Marne-la-Vallee Cedex, France) with 6 emitting lamps of 15 W/each emitting the light of 254 nm (irradiation time was optimized). After irradiation for a desired time period the fluorescence intensity was measured using fluorescence spectrometry (λ_ex_: 376 nm, gain: 100, emission wavelength from 400 to 700 nm measured by Synergy H1 (BioTek, USA)).

### Adsorption isotherms

Various concentrations of l-cysteine (5–30 µg/mL) were added to the MIP/NIP in order to investigate the interaction of the analyte with the MIP/NIP surface. The amount of l-cysteine isolated by the MIP (or NIP) was calculated according to the following equation:

1 $${\text{Q }} = \left( {{\text{C}}_{0} - {\text{ C}}_{{\text{e}}} } \right){\text{ V}}/{\text{m}}$$

where *Q* (mg/g) is the adsorbed mass of l-cysteine, *C*_*0*_ (mg/mL) and *C*_*e*_ (mg/mL) is the initial and equilibrium concentration of l-cysteine in the solutions, respectively, *V* (mL) is the volume of the sample solution, and *m* (g) is the mass of polydopamine layer in each plate. In each run, V = 50 µL and m = 8.4 × 10^−4^ g and 6.1 × 10^−4^ g for MIP and NIP, respectively.

Specific adsorption is one of the characteristics of MIPs. The specificity of MIP is often evaluated based on the partition coefficient, selectivity coefficient, and relative selectivity coefficient calculated by Eqs. (2)–(5):

2 $${\text{IF}} = {\text{Q}}_{{{\text{MIP}}}} /{\text{Q}}_{{{\text{NIP}}}}$$

3 $${\text{K}}_{{\text{d}}} = {\text{ Q}}/{\text{C}}_{{\text{e}}}$$

4 $${\text{K }} = {\text{ K}}_{{{\text{d1}}}} /{\text{K}}_{{{\text{d2}}}}$$

5 $${\text{K}}^{\prime} = {\text{ K}}_{{{\text{dMIP}}}} /{\text{K}}_{{{\text{dNIP}}}}$$

where *IF* is the imprinting factor, *K*_*d*_ is the distribution coefficient, *K* is the selectivity coefficient of MIP and NIP for l-cysteine, *K*_*d1*_ is the partition coefficient of l-cysteine, *K*_*d2*_ is the partition coefficient of competitors, and *K*ʹ is the relative selectivity coefficient^26^.

### Real sample analysis

In order to detect l-cysteine in the biological fluids, 1000 µL of fresh human urine was spiked with l-cysteine (100 µg/mL) and diluted with phosphate buffer (100 mM pH 7) to a final l-cysteine concentration of 30, 50, and 70 µg/mL. Next, tris(2-carboxyethyl) phosphine (TCEP) was added to a final concentration of 12.5 mg/mL and the mixture was shaken for 60 min at 37 °C. These samples were interacted with MIPs/NIPs at ambient temperature for 60 min. Finally, the procedure was carried out as mentioned in “Test sample analysis procedure” section. All the experiments were repeated in triplicates.

## Results and discussion

### The principle and the design of the study

**Figure 1:**
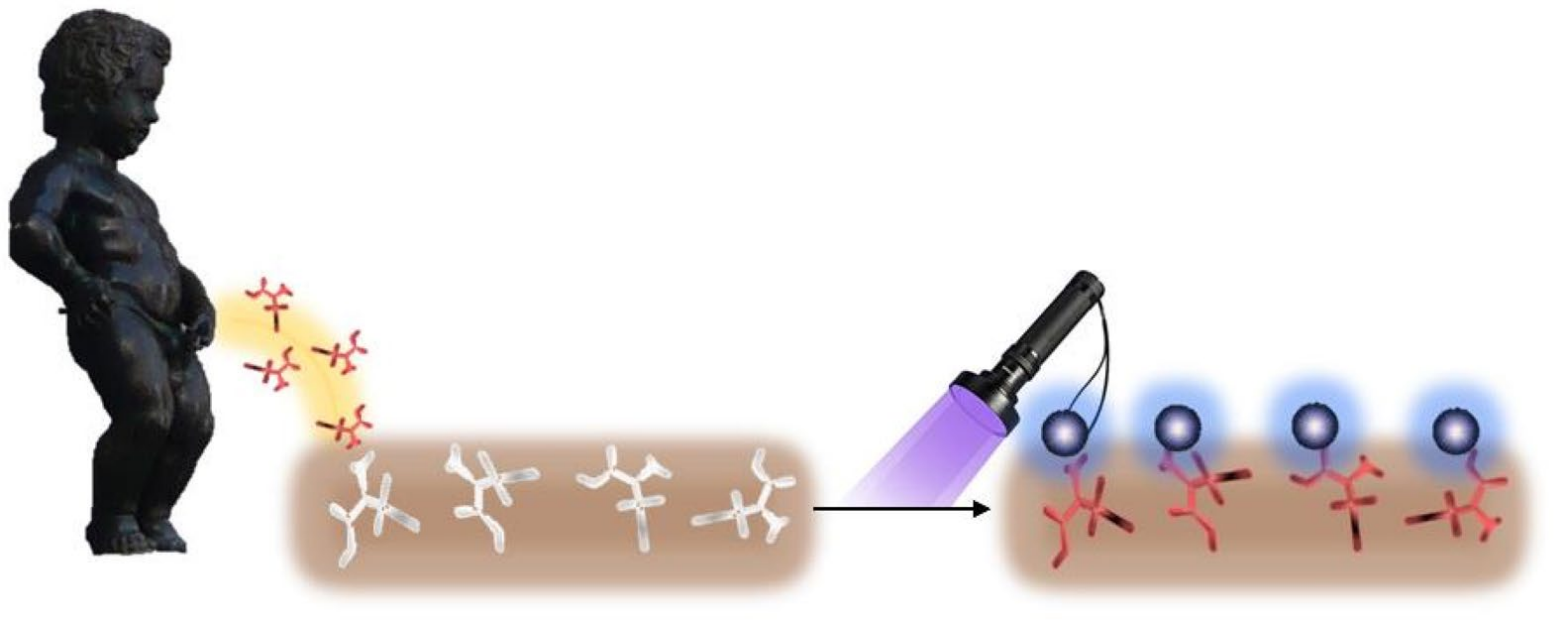
Schematic representation of overall approach.

**Figure 2:**
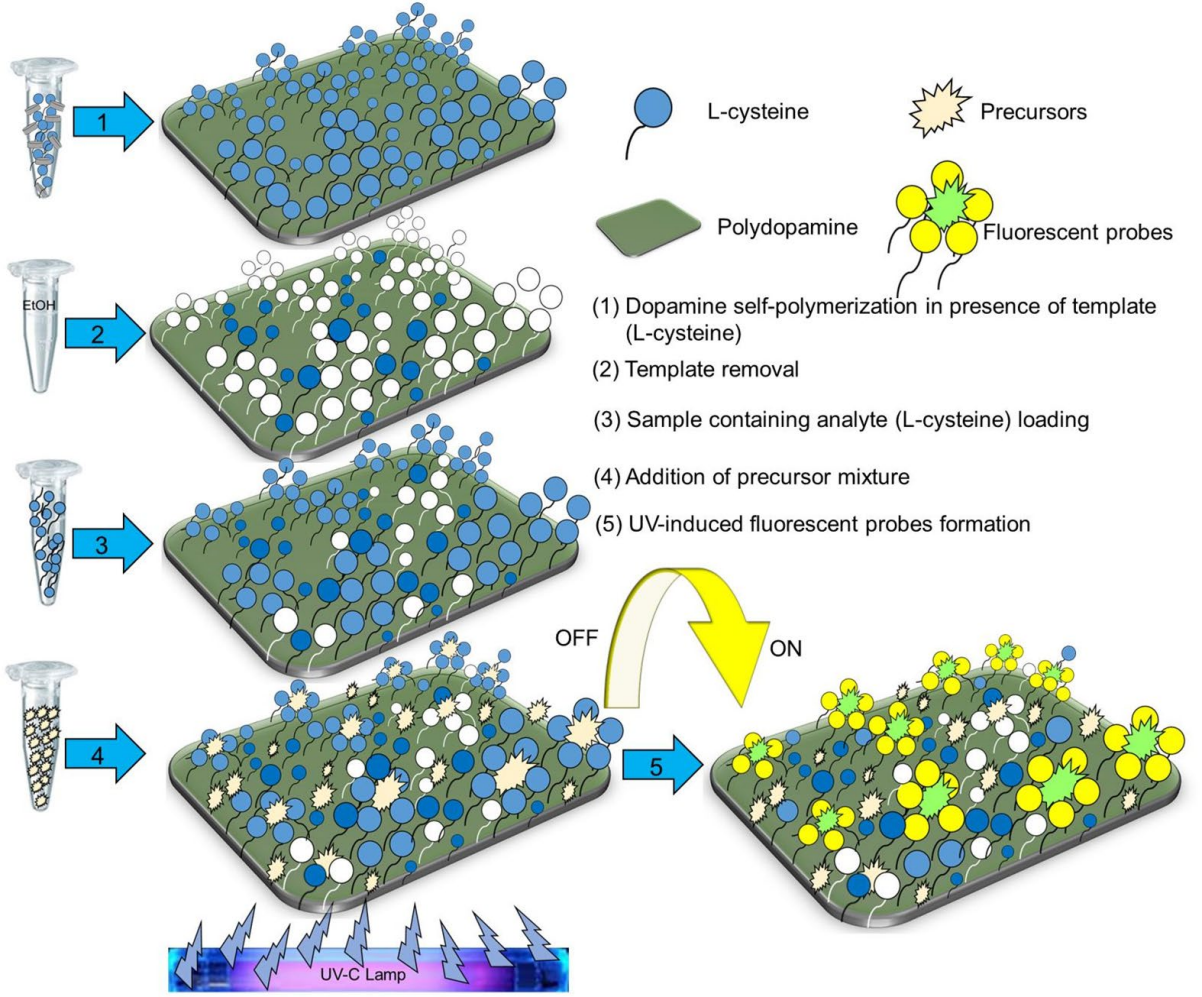
The principle and the process of the detection of thiols.

The schematic representation of the developed approach is shown in Fig. 1. In more details, the principle and the overall process of the detection of thiols consists of 5 steps as illustrated in Fig. 2: (1) The polydopamine MIP is polymerized in the presence of l-cysteine (template), (2) the template (l-cysteine) is removed and binding cavities are revealed, (3), the sample containing the analyte (l-cysteine) is loaded into the binding cavities of the prepared MIP layer, (4) precursor mixture (Zn^2+^ and Cd^2+^) is added, and (5) fluorescent probes are created by in-situ UV light-induced process. The intensity of fluorescence is proportionate to the amount of isolated l-cysteine as this thiol molecule serves as the capping agent of formed QD nanoparticles.

It is important to stress that this first-ever reported principle of detection of thiols rests in a unique in-situ UV light-induced formation of fluorescent QD nanoparticles developed in this research group^25,27^, and therefore is to a large degree an area with existing expertise. Similarly, polydopamine MIPs is also an area where our group has been active, and the materials were evaluated and characterized in our previous works^28^.

Given the steps in the process as shown in Fig. 2, the steps and experimental parameters within those that have to be optimized include the role of the monomer concentration in the MIP preparation, the template removal, the UV irradiation time in the QD cluster formation, followed by the l-cysteine determination performance parameters, and finally the determination of l-cysteine in urine. The results of investigations within these individual steps are then described in the following sections.

### Effect of monomer concentration and MIP thickness

**Figure 3:**
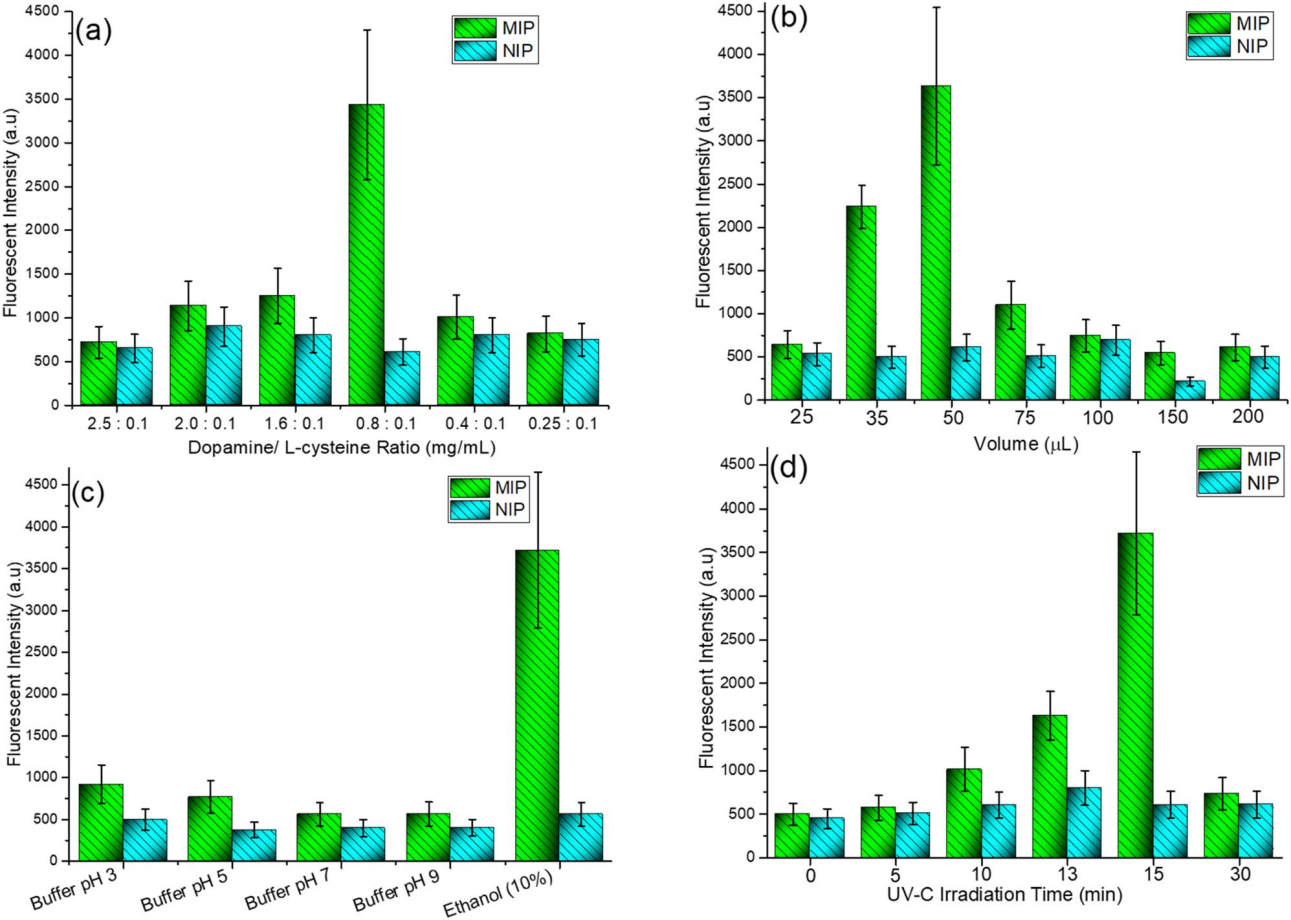
Optimization of key parameters, (**a**) Effect of different dopamine/l-cysteine ratio (50 µL polymerization mixture volume, 15 min UV irradiation, sample concentration 30 µg/mL), (**b**) Effect of polymerization mixture volume (Dopamine/l-cysteine ratio = 0.8/ 0.1, 15 min UV irradiation, sample concentration 30 µg/mL), (**c**) Effect of template removal solutions (2 × 50 µL), (Dopamine/l-cysteine ratio = 0.8/ 0.1, 50 µL polymerization mixture volume, sample concentration 30 µg/mL) and (**d**) the effect of UV irradiation time (dopamine/l-cysteine ratio = 0.8/ 0.1, 50 µL polymerization mixture volume, sample concentration 30 µg/mL).

The monomer-template ratio is a key parameter influencing the MIP efficiency; therefore different concentrations of dopamine (0.25–2.5 mg/mL) in the presence of 0.1 mg/mL l-cysteine (template) were tested. To evaluate the overall process, MIPs prepared with different monomer-template ratios were loaded with the fixed analyte (l-cysteine) concentration—30 µg/mL. After an incubation with the sample solution and washing (by 20 µL of water), the mixture of precursors (Zn^2+^ and Cd^2+^) was added, the plate was irradiated by UV light, and the fluorescence signal was acquired (λ_ex__=_ 376 nm and λ_em__=_ 442 nm). The same procedure was carried out with NIP in parallel. Considering the fluorescence intensity (Fig. 3a), the ratio of 0.8 mg/mL:0.1 mg/mL (dopamine:l-cysteine) was chosen as optimal. In case of the NIPs, a weaker fluorescence intensity compared to MIPs was detected (Fig. 3a). As NIPs represent the polymer without selective binding cavities, the fluorescent signal is caused by the nonspecific sorption of analyte molecules on the polymeric layer and should be treated as an undesirable background signal.

The film thickness of the MIP layer has a great effect on the sensitivity and adsorption mass^29^. Therefore, in the next step, different volumes of optimized polymerization mixture were tested to determine formation of the most efficient MIP layer within the used well plate. The results are shown in Fig. 3b. As demonstrated, the highest fluorescence intensity was achieved in case of the 50 µL volume. This volume was optimal from the layer thickness point of view as well as from the point of view of the uniform well bottom coverage. Lower volumes provided lower MIP thickness, while higher volumes, on the other hand, resulted in inefficient lengthy polymerization. This parameter was highly dependent on the well plate type and diameter of the wells. It is noteworthy that the NIP signal was significantly lower compared to MIP, which demonstrates that the nonspecific sorption of l-cysteine was almost negligible and, importantly, independent from the used volume.

### Template removal

The type of solvents used for the removal of the template molecules from the imprinted polymer is a critical parameter of the investigation. The washing solution should effectively disrupt the chemical or physical interactions between the template and the polymer but it should not cause any damage to the polymer leading to malfunction of the recognition cavities^30^. To investigate the effect of the eluent to remove of l-cysteine from MIPs, several buffers with pH range of 3–9 (100 mM, citrate–phosphate buffer pH 3, phosphate buffer pH 5 and 7, and borate buffer pH 9) and 10% (v/v) ethanol were selected. MIPs were washed twice by 50 µL of each eluent. To confirm the removal of l-cysteine from MIPs, after each rinsing, 2 drops of copper (II) sulfate (0.125 g/L) were added. The formation of a bluish-gray precipitate indicated residual l-cysteine within the MIP^31^. As shown in Fig. 3c, template removal by buffers with various pH provided results incomparable with the use of 10% ethanol.

### UV irradiation time in the QD cluster formation

As demonstrated in our previous study, UV light irradiation of the mixture of metal ions in presence of thiols led to formation of fluorescent nanoparticles^25^. This approach was exploited in this study taking advantage from the linear correlation between amount of thiol and fluorescence intensity of resulting fluorescent nanoparticles (i.e. in-situ formed fluorescent probes). To obtain the highest fluorescence signal, the UV light irradiation time was optimized. The fluorescence intensity was measured after 5–30 min of UV light irradiation using 30 µg/mL l-cysteine sample concentration (λ_ex_ 376 nm and λ_em_ 442 nm). As illustrated in Fig. 3d, fluorescence intensity increased from 5 to 15 min which is attributed to the interaction between l-cysteine and metal precursors leading to formation of Zn:Cd/S QDs. Sharp decrease of fluorescence intensity in case of longer irradiation times may be caused by chemical–physical behavior of dopamine. Polydopamine can generate hydroxyl radicals under UV light irradiation^32^. Hydroxyl radicals are highly reactive species that attack most of the organic molecules. They are highly oxidizing in nature, which is attributed to their oxidation potential^33^. Therefore, a significant decrease in fluorescent intensity after 15 min could be a result of l-cysteine degradation via hydroxyl radicals generated by polydopamine.

### The calibration curve for l-cysteine

**Figure 4:**
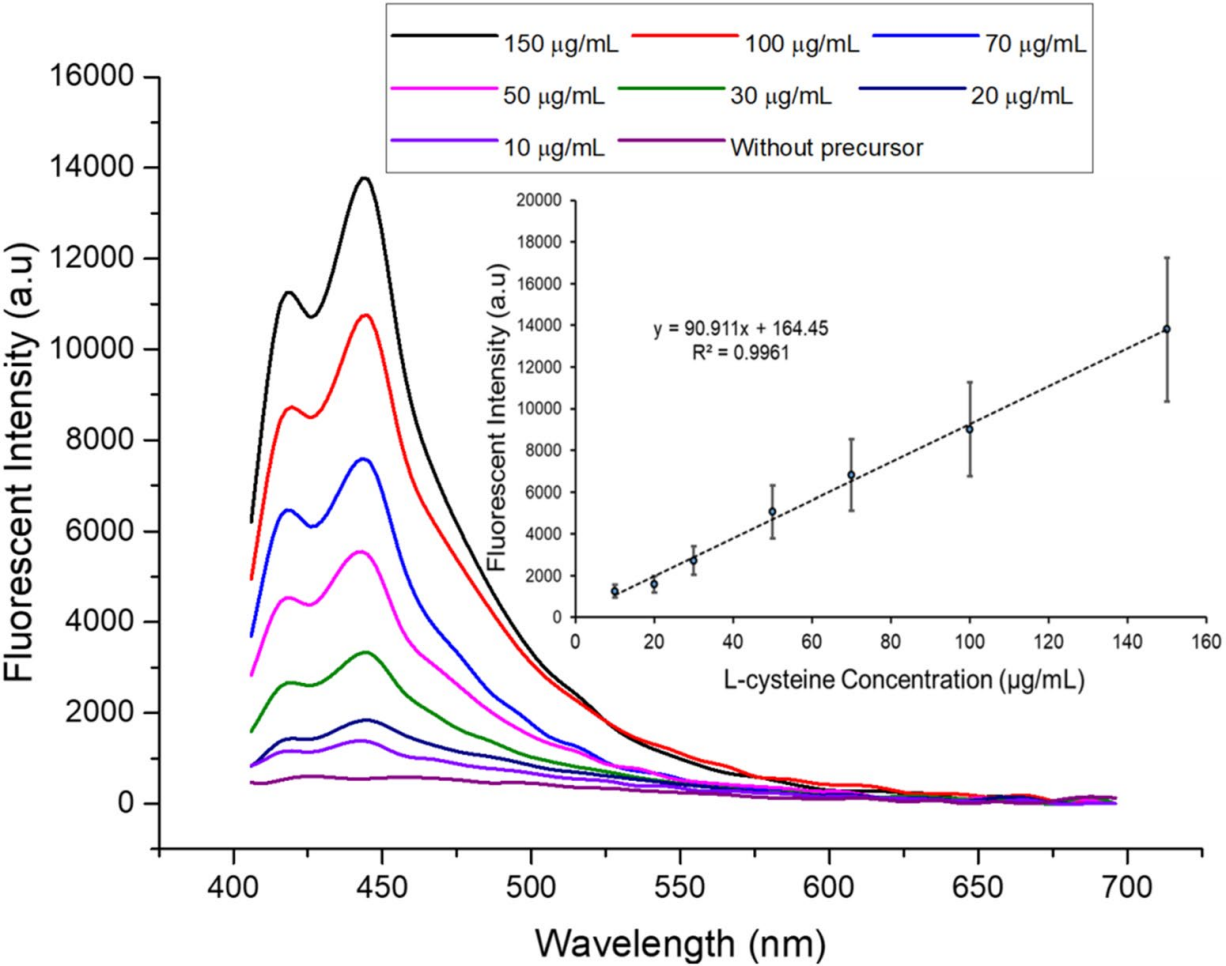
Fluorescence emission spectra of different concentrations of analyte (l-cysteine) within the sample analyzed by presented optimized procedure. Inset: linear response of calibration plot (Dopamine/l-cysteine ratio = 0.8/0.1, 50 µL polymerization mixture volume, 15 min UV irradiation, λ_ex_ 376 nm and λ_em_ 442 nm).

Under the optimized conditions, calibration curve was obtained using the proposed protocol. The calibration curve was linear over the concentration range of 12–150 µg/mL of l-cysteine. The linear equation was found to be y = 90.911x − 164.45, (R^2^ = 0.9961); where *y* is the fluorescence intensity (λ_ex_ 376 nm and λ_em_ 442 nm) and *x* is the concentration of l-cysteine in the sample. Fluorescence emission spectra and calibration curve are shown in Fig. 4. It is also shown that without addition of metal precursors, no fluorescence is observed as no QDs are formed. Broadening in the emission spectra is a possibility of vibrational relaxation from the covalent band in the presence of Sulphur within the l-cysteine^34^. Based on five repeated measurements, the limit of detection (LOD) and limit of quantitation (LOQ) for l-cysteine were found to be 3.6 and 12.1 µg/mL, respectively (LOQ = 10 Sb/m and LOD = 3Sb/m, where *Sb* is the standard deviation of the fluorescent intensity of blank solution and *m* is the slope of the calibration graph).

### Selectivity evaluation

**Figure 5:**
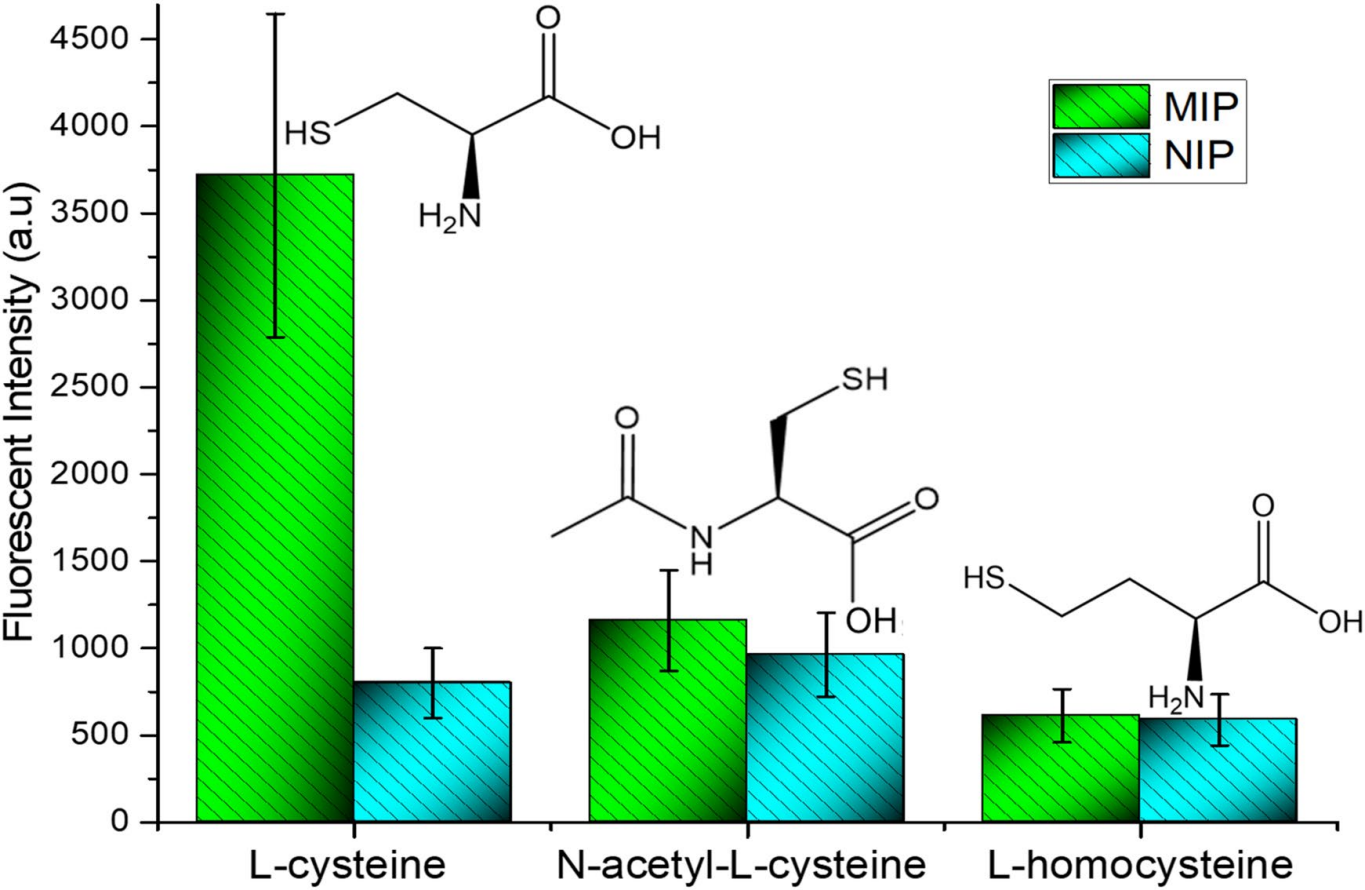
The fluorescence intensity of MIP/NIP for l-cysteine, N-acetyl-l-cysteine, and l-homocysteine (Dopamine/l-cysteine ratio = 0.8/ 0.1, 50 µL polymerization mixture volume, 15 min UV-C irradiation, analyte concentration 30 µg/mL).

One of the most important considerations for MIP application is the selectivity, so a detailed investigation of the selectivity was carried out. In brief, 30 µg/mL l-homocysteine, and N-acetyl-l-cysteine were selected as potential competitors to l-cysteine. The l-cysteine-selective MIPs were prepared as described above. Competitor molecules were loaded as the sample and after addition of metal precursors mixture and UV light irradiation, the fluorescence intensity was evaluated. As the competitor molecules did not interact with the polymeric layer, formation of fluorescent probes (i.e. ZnCd/S QDs) was impossible and only fluorescence at the level of nonspecifically adsorbed molecules was detected. The results are illustrated in Fig. 5. As shown, l-cysteine provided approximately sixfold and threefold greater fluorescence intensity compared to l-homocysteine and N-acetyl-l-cysteine, respectively. Considering the results, the prepared MIPs were significantly more selective for l-cysteine than the l-homocysteine and N-acetyl-l-cysteine. According to the fluorescence intensity, the MIPs exhibited the highest adsorption capacity for l-cysteine (1.73 mg/g), followed by adsorption capacity for N-acetyl-l-cysteine (0.45 mg/g) and l-homocysteine (0.17 mg/g).

Adsorbed mass (*Q*), distribution coefficients (*K*_*d*_), imprinting factor (*IF*), selectivity factors (*K*), and relative selectivity coefficient (*K*ʹ) are listed in Table 1.

**Table 1:** Adsorbed mass (Q), distribution coefficients (K_d_), imprinting factor (IF), selectivity factors (K), and relative selectivity coefficient (K′).

Sample	Q (mg/g)		K_d MIPs_	K_d NIP_	IF	K (l-cysteine/N-acetyl-l-cysteine)	K (l-cysteine/l-homocysteine)	K′
	MIPs	NIPs						
l-cysteine	1.73	0.26	1735.72	10.40	6.65	63.56	87.48	166.90
N-acetyl-l-cysteine	0.45	0.35	27.31	6.15	1.28			4.44
l-homocysteine	0.17	0.16	19.84	9.26	1.06			2.14

### Adsorption kinetics

**Figure 6:**
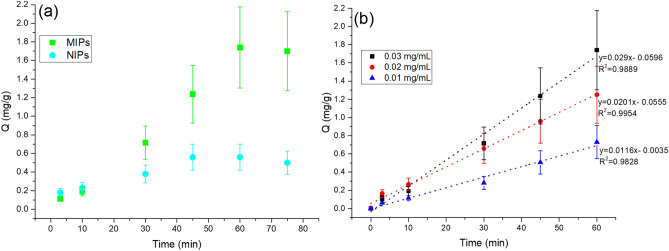
(**a**) Adsorption kinetics of MIP/NIP and (**b**) adsorption curves for analytes at different concentrations (10–30 µg/mL).

It can be seen from Fig. 6a that MIPs reached an equilibrium within 60 min. This is because the recognition sites produced by the surface imprinting are distributed on the polymer surface and therefore they are highly accessible and mass transfer resistance is low, which allow to achieve an adsorption equilibrium^26^.

At the same time, it is clear that the adsorption on the MIP surface was higher than that on NIP due to the presence of specific sites on the polydopamine surface. Generally, the value of adsorbed mass (Q) on the adsorbent was calculated from the analyte remaining in the solution (C_0_ − C_t_)^30,35,36^. Relationships between the contact time and l-cysteine adsorption at different concentrations is shown in Fig. 6b. As is illustrated, there is a linear adsorption in the range of 10–30 µg/mL up to the equilibrium time.

**Figure 7:**
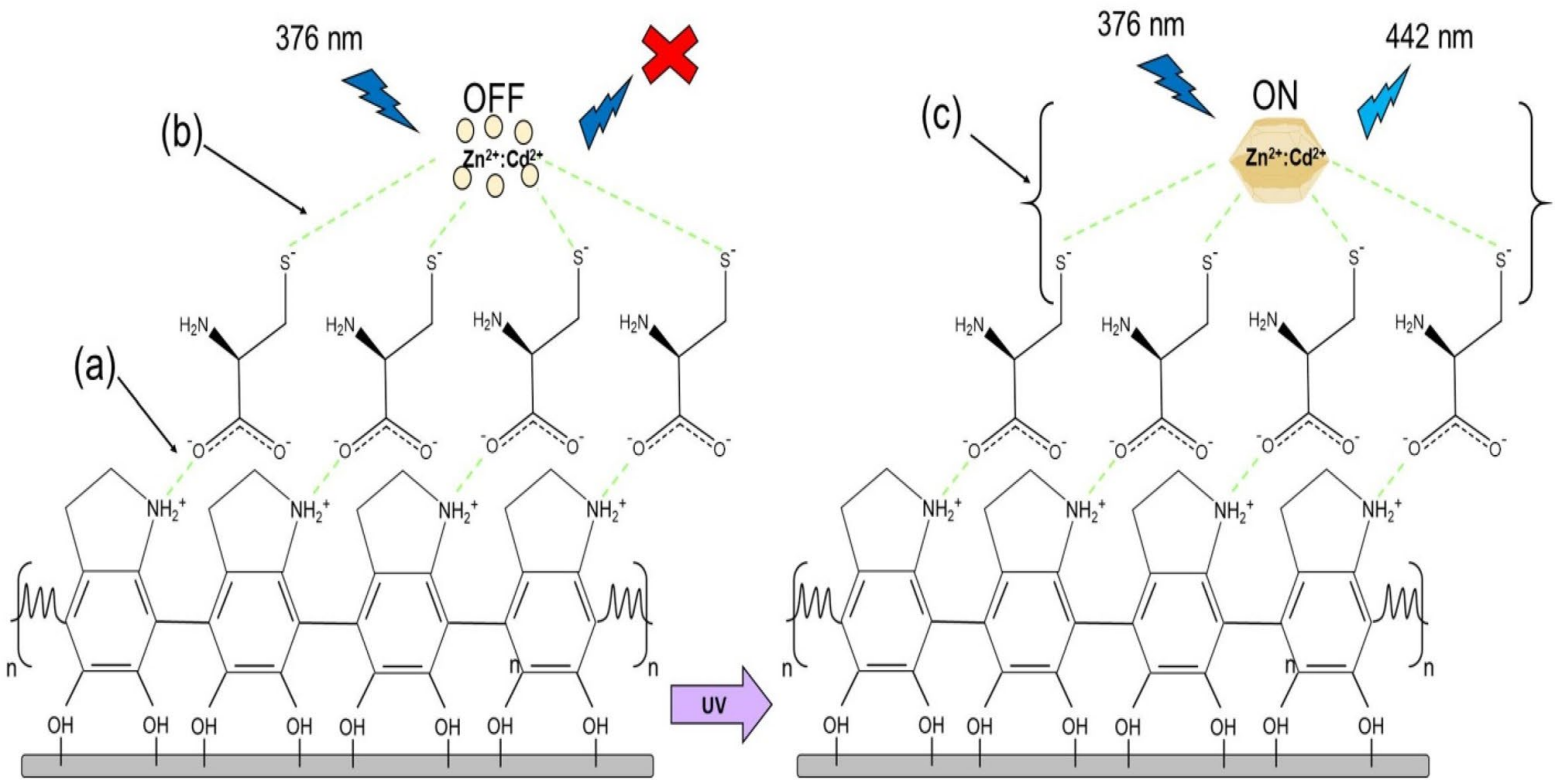
(**a**) Schematic illustration of the possible situation in polydopamine and l-cysteine interaction, (**b**) thiolate anion interaction with the precursors, and (**c**) formation of ZnCd/S QDs after UV irradiation.

In this work, dopamine self-polymerization in presence of l-cysteine took place in Tris–HCl buffer pH 8.5. According to the l-cysteine pKa values (1.9, 8.2, and 10.3), its chemical forms depend on pH of the medium. Hence, in the pH around 8, electrostatic bonds take place between polydopamine NH_2_^+^ and l-cysteine COO^¯^^37,38^. Scheme of interactions taking place during self-polymerization of dopamine in the presence of l-cysteine is illustrated in Fig. 7a. The l-cysteine thiol group can participate in nucleophilic or Michael additional reactions. The relative reactivity of thiol (SH) and thiolate anion (S^¯^) of l-cysteine with pKa (6.4, 7.4, or 8.4) are 10, 50, 90 and 90, 50, 10%, respectively. Owing to the fact that mixture of precursors was prepared at pH 7, the thiolate-precursor interaction rate increases as precursors are probably surrounded by thiolate anions^39,40^. Thiolate anion interaction with precursors is illustrated in Fig. 7b. The precursors were in OFF-form (only metal ions) and therefore under excitation by 376 nm, no fluorescence emission was observed.

After exposure to UV light, the precursors converted into ZnCd/S QDs (ON-form) providing fluorescent signal with emission at 442 nm after excitation by 376 nm. The formation of ZnCd/S QDs after UV irradiation is illustrated in Fig. 7c.

### Detection of l-cysteine in real samples

It should be noted that normal cystine excretion is 30 mg/day. Patients suffering with cystinuria normally exceed 400 mg/day excretion. The conventional test for measuring urinary cysteine levels is the sodium cyanide-nitroprusside test. In this test, cyanide converts cystine to cysteine, which then binds to the nitroprusside creating an intense purple color in just a few minutes; but it is noteworthy that this test is typically positive when cystine levels are higher than 75 mg/L^41,42^. The utilization of the here presented method for real sample analysis is an important result of this work. The recovery test was carried out by the standard addition method to evaluate the developed strategy with the optimized parameters in the human urine. However, due to the disulfide bonds formed between cysteine molecules, no significant fluorescence was achieved. The normal pH range of urine is 4.5–7.8^43^. In the pH lower than 7.4, more than 50% of l-cysteine is in the thiolate anion form, which is more prone for oxidation and formation of disulfide bond^39,44^. Hence, the majority of l-cysteine is in the form of l-cystine lacking the thiol group. Therefore, it has no ability to react with the metal precursors leading to fluorescent probes (i.e. QDs) formation. One of the suitable reductants for cleavage of the disulfide bond is TCEP^45^. Accordingly, after l-cysteine standard addition to the human urine, the samples underwent a reduction in the presence of TCEP. The achieved results are shown in Table 2. The values found for samples were in the range of 18.73–89.56 µg/mL, with relative standard deviations (RSD %) ranging from 0.01 to 12.90%. The comparison between this method and conventional methods has been mentioned in Table 3. Even though, some methods may reach lower limits of detection, we would like to stress that these methods may possess significant disadvantages including time-consuming and costly protocols, expensive instrumentation or limited expiration time. Moreover, the amounts of cysteine excreted in case of cysinuria patients is relatively high and more convenient method might be beneficial.

**Table 2:** Determination of l-cysteine in human urine samples (n = 5).

Sample	Added concentration (µg/mL)	Found (µg/mL)	RSD (%)
1	0	18.73 ± 3.07	16.39
2	30	49.04 ± 1.14	2.32
3	50	70.32 ± 6.11	8.69
4	70	89.56 ± 11.55	12.90

**Table 3:** Comparison between this method and conventional methods.

Method	Advantage	Disadvantage	LOD (µg/ml)	Price ($)	Ref
HPLC	High accuracy and reproducibility	Expensive and sophisticated instruments	~ 0.01	> 20,000	^46,47^
		Time consuming			
		Derivatization required			
Cysteine assay kits	Simple and sensitive	Storage at − 20 °C	1.2	≥ 530	^48,49^
		Expensive enzymes required			
		Limited expiration			
Sodium nitroprusside test	Simple and fast	Imperfect in accuracy	75	200	^50^
		Depend on cystine solubility			
		Do not distinguish cystine from soluble thiol drug-cysteine complexes			
This method	Simple, fast and sensitive	UV light required	3.6	≤ 20	^–^

## Conclusions

In this study, a new approach to the detection of thiols in liquid samples was explored and a method for the fluorescence detection of l-cysteine was developed. Importantly, even though the benefits of the method were demonstrated by the determination of l-cysteine in urine, its simplicity as well as variability enable its easy adjustment for the detection of any other thiol in liquid samples (e.g. biological fluids). The main strengths of this approach and the method based upon it include the selectivity given by MIP-based approach and sensitivity given by fluorescent labeling of the analyte using the simple and efficient in-situ UV light induced formation of fluorescent probes. Moreover, this strategy requires only minimal sample and reagents volumes, incomparably smaller than those commonly used in chromatographic techniques. Finally, the potential for miniaturization in a form of a portable or even hand-held device can be envisioned, especially taking into the consideration the advances in light emitting diode light sources or detection components (e.g. photodiodes, miniaturized photomultipliers).
